# Spatial modelling of contribution of individual level risk factors for mortality from Middle East respiratory syndrome coronavirus in the Arabian Peninsula

**DOI:** 10.1371/journal.pone.0181215

**Published:** 2017-07-31

**Authors:** Oyelola A. Adegboye, Ezra Gayawan, Fahad Hanna

**Affiliations:** 1 Department of Mathematics, Statistics and Physics, College of Arts and Sciences, Qatar University, 2713 Doha, Qatar; 2 Department of Statistics, Federal University of Technology, Akure, Nigeria; 3 Department of Public Health, College of Health Sciences, Qatar University, 2713 Doha, Qatar; UNITED KINGDOM

## Abstract

**Background:**

Middle East respiratory syndrome coronavirus is a contagious respiratory pathogen that is contracted via close contact with an infected subject. Transmission of the pathogen has occurred through animal-to-human contact at first followed by human-to-human contact within families and health care facilities.

**Data and methods:**

This study is based on a retrospective analysis of the Middle East respiratory syndrome coronavirus outbreak in the Kingdom of Saudi Arabia between June 2012 and July 2015. A Geoadditive variable model for binary outcomes was applied to account for both individual level risk factors as well spatial variation via a fully Bayesian approach.

**Results:**

Out of 959 confirmed cases, 642 (67%) were males and 317 (33%) had died. Three hundred and sixty four (38%) cases occurred in Ar Riyad province, while 325 (34%) cases occurred in Makkah. Individuals with some comorbidity had a significantly higher likelihood of dying from MERS-CoV compared with those who did not suffer comorbidity [Odds ratio (OR) = 2.071; 95% confidence interval (CI): 1.307, 3.263]. Health-care workers were significantly less likely to die from the disease compared with non-health workers [OR = 0.372, 95% CI: 0.151, 0.827]. Patients who had fatal clinical experience and those with clinical and subclinical experiences were equally less likely to die from the disease compared with patients who did not have fatal clinical experience and those without clinical and subclinical experiences respectively. The odds of dying from the disease was found to increase as age increased beyond 25 years and was much higher for individuals with any underlying comorbidities.

**Conclusion:**

Interventions to minimize mortality from the Middle East respiratory syndrome coronavirus should particularly focus individuals with comorbidity, non-health-care workers, patients with no clinical fatal experience, and patients without any clinical and subclinical experiences.

## Introduction

Middle East respiratory syndrome coronavirus (MERS-CoV) is a respiratory contagious pathogen that is contracted via close contact with an infected subject [1]. MERS-CoV transmission had been known to occur via animals-to-humans; however, subsequent cases of human-to-human transmission have resulted in households and health care facility outbreaks have been documented [2–8]. The disease appears to have been transmitted from camels to humans, and recent studies have revealed an association among the virus found in humans with that found in camels [9]. Additionally, some studies have found antibodies to the virus in camels located in Africa and the Middle East [10]. In 2012, dozens of cases of people infected with MERS-CoV were reported in the Kingdom of Saudi Arabia (KSA) [9]. It has been reported that the disease has been fatal in 40% of confirmed cases [11].

Outbreaks of the disease have exposed the general populace, particularly health-care workers, in different countries to a greater risk, especially in the Arabian Peninsula where most of the cases have been reported. The epidemic has serious public health implication. It is suspected that people with pre-existing chronic medical conditions (comorbidities) are more prone to being infected by the illness or to developing a severe case resulting in fatality [12]. Patients with chronic diseases such as diabetes, chronic lung disease and heart conditions especially older males are at higher risk [13, 14]. Strong links between health-care facilities and the outbreak of the disease has also been found in Jeddah, where the majority of patients were in contact with other patients or health-care workers [15]. Elsewhere, the transmission of MERS-CoV in household contacts revealed that an outcome of approximately 5% as the rate of secondary transmission occurred at home [12].

The Centers for Disease Control and Prevention indicated that most index case-patients have either resided in, or have travelled to areas neighboring the Arabian Peninsula, specifically, Saudi Arabia, the United Arab Emirates, Qatar, Jordan, Oman and Kuwait [16, 17]. Although the outbreak has been mostly within the Arabian Peninsula, and concentrated in major cities and towns, a few cases have been reported in western countries and more recently, in South Korea [18]. The epidemic has understandably caused serious travel panic among the general public as well as among health-care workers and policy makers worldwide. Going by the number of new cases of international occurrence of MERS-CoV outside the Arabian Peninsula [19], the question that should be on everyone’s mind right now is, especially since the disease was reported in South Korea, is what is the likelihood of similar outbreaks in countries in close proximity with the origin of the disease?

Most MERS-CoV cases reported have probably acquired infection through human-to-human transmission [14]. Among 144 confirmed and 17 probable cases analyzed by the MERS-CoV Research Group in November, 2013, 95 (59%) were classified as secondary cases with epidemiological links to other confirmed cases [20]. Among these, most acquired the infection in health-care settings (63.2%), followed by those infected in household settings (13.7%) [20].

The epidemiologic features of the disease are difficult to determine with the currently available information. The analyses of the disease outbreaks will be a versatile tool for studying and understanding transmission and spread of the disease. It will be useful in cubing its upsurge, and possibly its containment or eradication. Yesterday, it was AIDS, today Ebola, MERS-CoV and Zika. What will it be tomorrow? It is, therefore, a matter of urgency to examine the likelihood of fatality as a result of MERS, keeping in mind the associations of individual- and work-related risk factors with the disease. The present paper aims to use geoadditive regression model [21] to elucidate the epidemiological risk factors and geographical distribution of the transmission and severity of the outbreak. Specifically, we investigated the effect of comorbidity and other individual- and work related- level risk factors including the geographical spread of mortality from MERS-CoV across the regions of KSA.

The motivating dataset for this study is introduced in section 2, while Section 3 presents the modeling technique. The results and discussion of the findings are presented in section 4 and 5 respectively. Findings from this study will help public health practitioners, policy makers and program managers monitor and design intervention strategies aimed at minimizing deaths due to the Middle East Respiratory Syndrome Coronavirus in the Arabian Peninsula.

## Materials and methods

### Data sources

**Fig 1:**
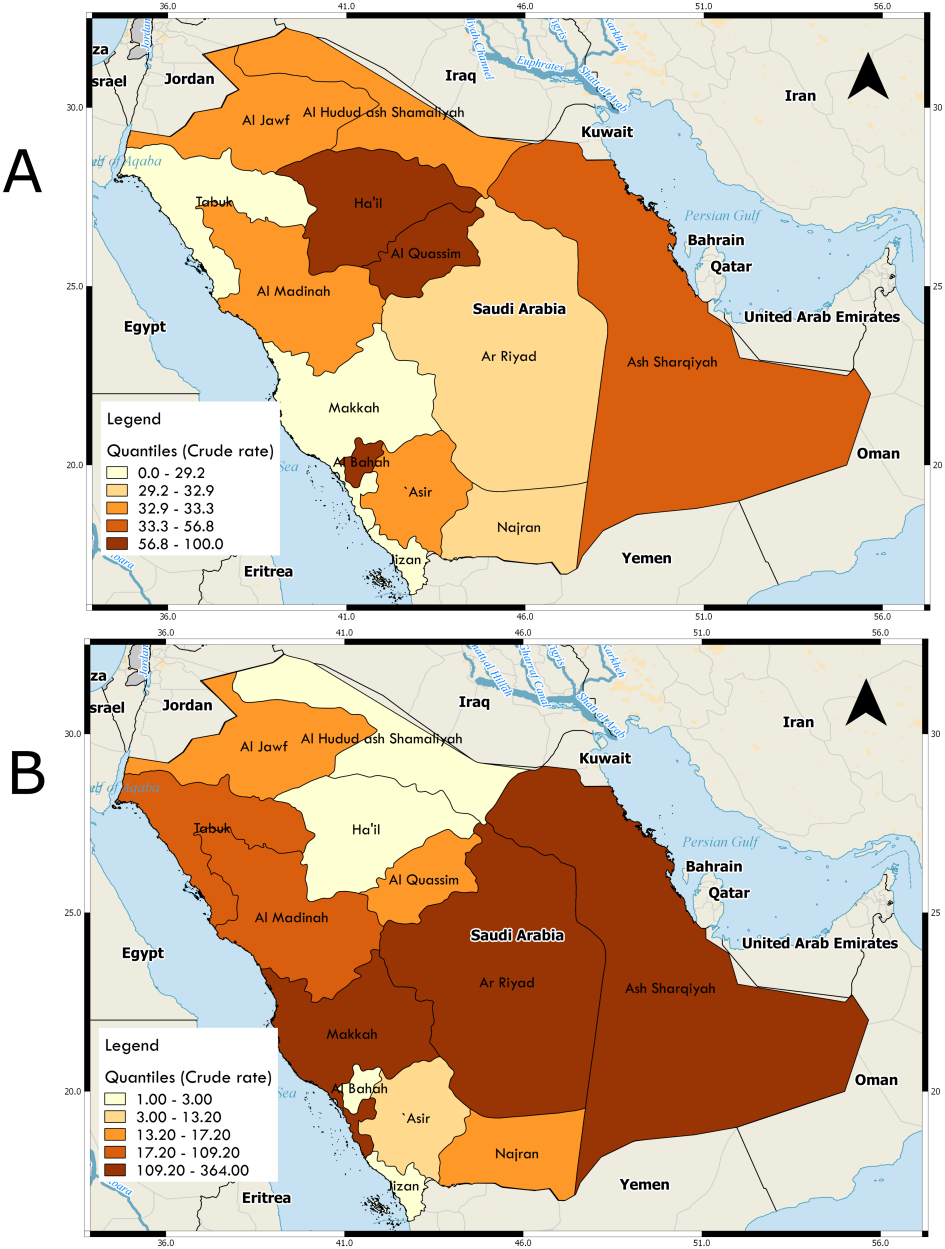
Map of Kingdom of Saudi Arabia showing the distribution of (a) the number of MERS cases in the 13 regions of Saudi Arabia, (b) the crude mortality rate for MERS-CoV disease. The maps are based on regional aggregated counts of MERS cases over the study period.

This study was based on a retrospective data on the Middle East respiratory syndrome coronavirus (MERS-CoV) outbreak in the Kingdom of Saudi Arabia (KSA) between June 6, 2012 and July 17, 2015. The data set was the case-by-case data list compiled and regularly maintained by Dr. Andrew Rambaut [22] from various sources including World Health Organization(WHO) bulletins, Ministry of Health of the Kingdom of Saudi Arabia and media reports. MERS-CoV cases were confirmed via real-time RNA-positive using Reverse transcription polymerase chain reaction (RT-PCR) showing positive PCR on at least two specific genomic targets upstream E protein (upE) and ORF1a or a single positive target (upE) with sequencing of a second target (RdRpSeq assay) or N gene (NSeq assay) [23]. See Fig 1 for the map of the crude rates and counts of infected MERS cases across the KSA created from case-by-case data.

The outcome of interest in this study is the survival status of the infected individual (dead/alive). The survival status of an infected individual is determined by whether the individual is dead or alive at the time of reporting [22]. Based on available data and recent literature [24], the following characteristics were used as individual level risk: age (in years) and gender, clinical outcome, region of infection, history of contact with animal, history contact with camels, whether the patient is a health-care worker (including all personnel that work in a health-care facility), presence or absence of any comorbidities in a patient, where or through who the patient contracted the disease (if known) and whether the patient is a primary contact (the first case within a defined group) or a secondary contact (individual infected by primary contact). The region of residence of the respondents was geo-referenced and used for the spatial analysis. Table 1 presents the frequency distribution of the recorded cases based on the variables considered.

**Table 1:** Risk factors analyzed in MERS-CoV mortality data with chi-square test.

Risk factors	Category^‡^	% Dead^†^	*χ*^2^ *p* − *value*
Gender	Male (65.7%)	28%	0.0259
	Female (32.6%)	36%	
	NA (1.8%)		
Comorbidity	Present (52.7%)	46.7%	< 0.0001
	Absent(47.3%)	17.1%	
Comorb-cat	None (47.3%)	17.1%	< 0.0001
	One (14.8%)	14.8%	
	More than one (37.9%)	49.0%	
Animal Contact	Yes (8.9%)	38.8%	0.2215
	No (91.1%)	32.3%	
Camel Contact	Yes (7%)	37.3%	0.4228
	No (93%)	32.5%	
Health Care Worker	Yes (13%)	8.6%	< 0.0001
	No (87%)	36.6%	
Clinical	Fatal (21.2%)	94.6%	< 0.0001
	Clinical (60.6%)	20.6%	
	Subclinical (15.8%)	1.3%	
	NA (2.2%)		
Where-contracted	Family or Community 19.1%	40.4%	< 0.0001
	Hospital (11.6%)	44.6%	
	HCW (2.7%)	7.7%	
	No Contact (23.4%)	18.8%	
	NA (38.2%)		
Secondary Contact	Yes (40%)	25.7%	0.0001
	No (60%)	37.6%	
Ageat onset	Median = 52years (IQR = 37- 65)		< 0.0001

^‡^: Percentage based on number of infected individuals within each category.

^†^: The percentages were calculated based on the number of deaths within each category.

(For example, 36% of infected females died of the disease).

### Exploratory analysis

Firstly, univariate analyses were carried out to explore the relationship between the patient survival status and several risks and demographical factors using SAS 9.3 [25]. We present the frequency of risk factors and survival status as percentages of deaths within each category (Table 1). To identify associations between categorical risk factors and survival status of MERS-CoV disease, we used Pearson’s chi-square statistics for testing independence in contingency tables [26]. The chi-square test measures how “close” the observed values are to those which would be expected under the fitted model.

Similarly, local spatial heterogeneity of MERS disease was evaluated in SaTScan [27]. SaTScan is widely used for local cluster detection, which is good for detecting large clusters as well as to evaluate outliers when the outlier pattern is very strong or a small maximum search window is used [28]. The idea of Poisson model based SaTScan circular version is to recognize sets of regions where the disease count is significantly larger than expected [29]. SaTScan’s Poisson log likelihood ratio statistics was applied to regional aggregated MERS counts in circular windows of increasing radius centered at each region centroid with a maximum cluster size of provinces covering 50% of the national population. Clusters with the largest test statistics were tested for statistical significance. This significance was assessed using the default 999 Monte Carlo trials drawn under the null hypothesis that the observed case count represents the census distribution. If the p-value derived by ranking a test statistic calculated from observed data against the 999 statistics calculated similarly for the Monte Carlo trials was below our alpha level of 5%, then the observed cluster was considered significant [27–29]. Additionally, the Wang’s *q*-Statistics [30, 31] was used to test the global stratified spatial heterogeneity of occurrence of MERS disease. In all analyses, p-values of less than 0.05 were considered statistically significant in all tests.

### Statistical analysis

Our approach to spatial analysis is based on the framework of structured additive regression model [21]. Geoadditive Bayesian models have been used and described in details in several studies [32–34]. In brevity, suppose *y*_*i*_ is the survival status of an infected individual *i* at location *s*_*i*_ and *υ* is a vector of observed covariates, which could be categorical or continuous. We define *y*_*i*_ = 1 indicating the individual die of MERS disease or *y*_*i*_ = 0 otherwise. *y*_*i*_ is assumed to have a binomial distribution given as:

$$\begin{array}{c} y_{i}=Bin\left(n_{i},p_{i}\right) \end{array}$$ (1)

where the probability “*p*_*i*_” of dying from the infectious disease is given as:

$$\begin{array}{c} p_{i}=P\left(y_{i}=1\right)=\frac{exp(\eta_{i})}{1+exp(\eta_{i})} \end{array}$$ (2)

The predictor indicator “*η*_*i*_”, is a known response function with a logit link function as specified in Eq 3 [32]. The influence of the covariates can be modelled assuming a logit link function on the proportion.

To be able to incorporate spatial covariate and to model the continuous variable, age using smooth function, we adopt the logistic model with structured additive predictors defined as:

$$\begin{array}{c} \eta_{i}=logit\left(p_{i}\right)=log\{\frac{p_{i}}{1-p_{i}}\}=f\left(x\right)+f_{geo}\left(s_{i}\right)+\upsilon^{\prime }\beta \end{array}$$ (3)

where *f*(*x*) is a nonlinear effect smooth function assumed for age, *f*_*geo*_(*s*_*i*_) is the geographical effect, and *β* is a vector of fixed effect parameters for the categorical covariates. The predictor will be of the form *η*_*i*_ = *β*_1_ ⋅ Comorbidity + … + *β*_7_ ⋅ Clinical + *f*_1_(*age*) + *f*_*geo*_(*region*). We also included an interaction term between comorbidity and age and modeled that using smooth function. The aim was to examine how comorbidity varies smoothly across age (The results of this model are presented in Table 2).

**Table 2:** Posterior odds ratio and 95% credible interval of the effect of various categorical variables on mortality due to MERS-CoV.

Variable	Posterior odds ratio	Credible interval
Comorbidity (Present vs Absent)	2.071	1.307, 3.263
Animal contact (Yes vs No)	1.634	0.527, 4.847
Camel contact (Yes vs No)	0.741	0.229, 2.534
Health Care Worker (Yes vs No)	0.372	0.151, 0.827
Secondary Contact (Yes vs No)	1.089	0.696, 1.658
Sex (Male vs Female)	1.197	0.787, 1.821
Clinical		
Clinical vs Fatal	0.040	0.025, 0.063
Subclinical vs Fatal	0.004	0.001, 0.016

Parameters estimation follow from the Bayesian context whereby all parameters and functions are considered as random variables and appropriate priors are assumed. Independent diffuse priors are assumed to estimate the categorical covariates. For the smooth function for the nonlinear effects of age, Bayesian P-splines prior was assumed [35, 36]. Following [35–37], the P-spline assumes that the spline can be written as a linear combination of basis functions (B-spline: *B*_*j*_), denoted by:

$$\begin{array}{c} f_{j}\left(x_{j}\right)=\sum_{j=1}^{j}\beta_{j}B_{j}\left(x_{j}\right) \end{array}$$

The *β*_*j*_ are unknown regression coefficients that can be defined to follow a first or second order random walks smoothness *β*_*j*_ = 2*β*_*j*−1_ − *β*_*j*−2_ + *u*_*j*_ with Gaussian errors $u_{j}∼N\left(0,\tau_{j}^{2}\right)$. The smoothness of *f* is control by the variance parameter $\tau_{j}^{2}$, which is also considered as a random variable and a highly dispersed inverse gamma prior assumed for the variance, $\tau_{j}^{2}∼IG\left(a_{j},b_{j}\right)$. This way, it is jointly estimated with the regression coefficients [36].

The spatial effects *f*_*geo*_(*s*_*i*_) = *β*_*geo*,*s*_ was modeled assuming a Gaussian Markov random field prior [36, 38] defined by:

$$\begin{array}{c} \beta_{geo,s}|\beta_{geo,u},u\neq s∼N(\sum_{u\in \partial_{s}}\frac{1}{N_{s}}\beta_{geo,u},\frac{\tau^{2}}{N_{s}}) \end{array}$$ (4)

where *N*_*s*_ is the number of adjacent regions, and ∂_*s*_ denotes the regions which are neighbors of region *s*. This defines areas as neighbours if they share a common boundary. The spatial variance was also assigned an inverse Gamma prior.

Sensitivity to the choice of hyper-priors was investigated by varying the values of *a*_*j*_ and *b*_*j*_. The results turned out to be indistinguishable. Findings reported are based on *a*_*j*_ = *b*_*j*_ = 0.001. The posterior distribution is intractable so, Markov chain Monte Carlo (MCMC) algorithm was adopted to generate sample from the posterior distributions, which allows for estimation and inference to be made for all parameters. The posterior odds ratios (OR) and their 95% confidence intervals (95% CI) were calculated using BayesX version 2.1 [39, 40].

## Results

### Exploratory data analysis

Table 1 presents the summary profile characteristics and univariate analysis of the categorical variables in the dataset and age. 959 MERS cases were recorded in KSA during the study period with 317 (33%) deaths while 67 (7%) had contact with camels or camel products, 126 (13%) were health-care workers and 52.7% had some kind of comorbidity (Table 1). Similarly, out of the 630 male patients, 28% died as a result of MERS-CoV while only 36% of the females died from the disease (Table 1). The median age for males was 53.5 years (interquartile range 39-66) while the median age for females was 48 years (interquartile range 32-63).

Not all of the comorbidities were equally prevalent. While most of the patients in this study had some kind of underlying comorbidities (52.7% have at least one comorbidities), around 38% of all patients had more than one comorbidities with the most common being obesity, diabetes and hypertension (which occurred in more than 50% of those with any underlying comorbidity) (Table 1). Others comorbidities were heart disease, respiratory disease, pneumonia, renal/kidney disease and asthma.

Pearson’s chi-square test of health outcomes between subgroups shows significant difference in gender, comorbidity, health-care worker, clinical outcome, contact type and secondary contact (Table 1). About 3 out of every 10 males died of MERS disease, compared to 28% of the females. The percentage of health-care workers that died of MERS (8.73%) were much less than non-health care workers (36.5%), while 46.14% of persons with comorbidity died of MERS compared with 17.05% of those without comorbidity. Similarly, there effect of comorbidity on mortality from MERS-CoV was significant; patients who died of the disease were more likely to have one or more comorbidities with an odd ratios of 3.4 and 4.7 respectively.

**Fig 2:**
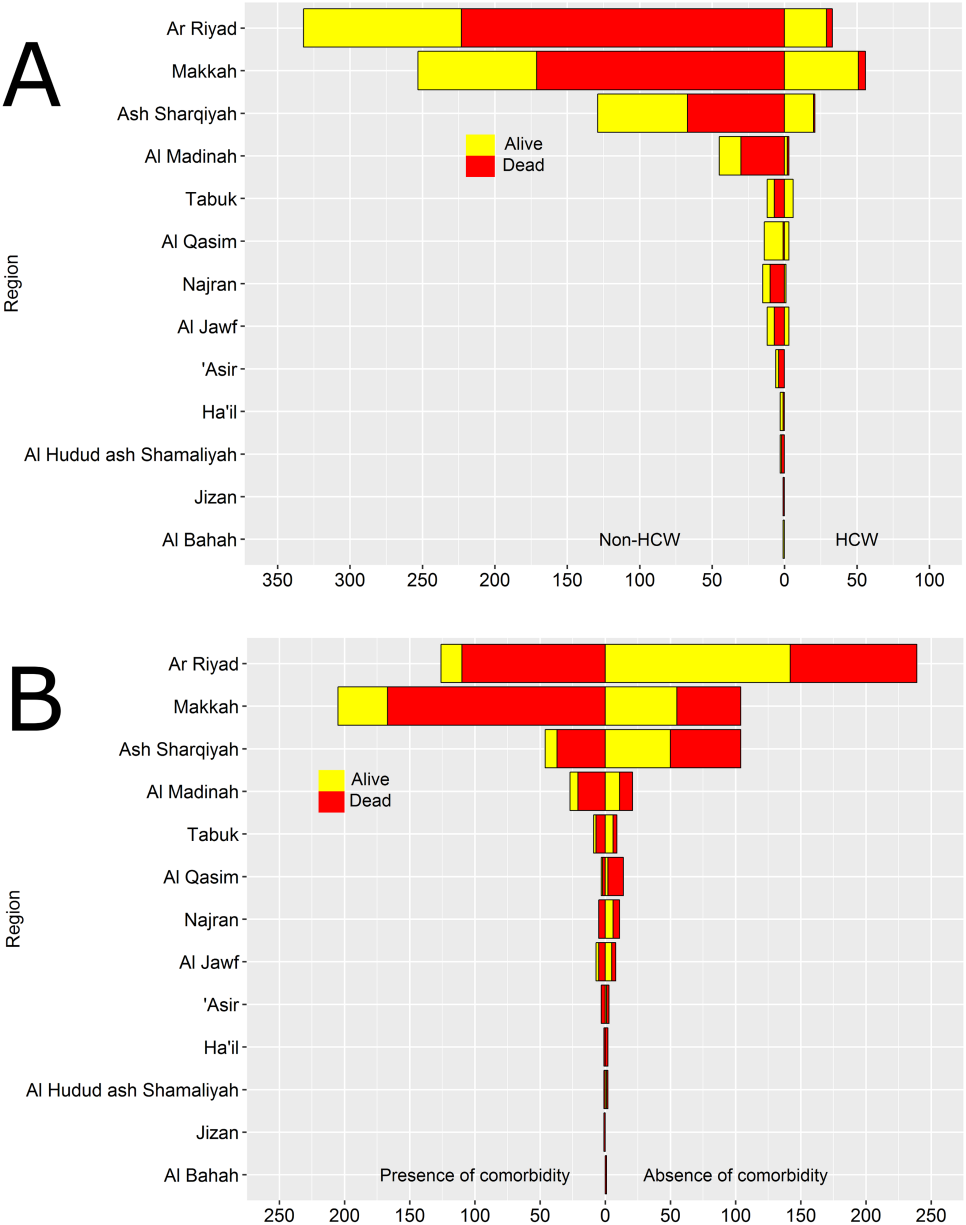
Pyramids showing the distribution of mortality among infected individuals with some kind of comorbidity (top) and health-care workers (HCW, bottom), in the 13 regions of Saudi Arabia. The horizontal axis represents the number of MERS cases.

Fig 1 shows the study area and the distribution of the number of infected people and the number of people who died of the disease in the 13 provinces of the KSA. Most of the MERS cases occurred in Ar Riyad (38%) and Makkah (34%) provinces. Fig 2 shows the pyramids of the distribution of the mortality status for the 13 regions based on comorbidity status (upper part) and whether or not the individual was a health worker (lower part). From the pyramids, it is clear that the highest number of cases occurred in Ar Riyad followed by Makkah. The incidence of comorbidities was significantly higher among patients in Ar Riyad, Makkah and Ash Sharqiyah (about half of the cases of comorbidities occurred in these three regions). Al Bahah had the least cases of infected individuals. Similarly, Ar Riyad, Makkah and Ash Sharqiyah recorded the highest number of infected health-care works (Fig 2 bottom). The proportion of health-care workers who died of MERS-CoV were smaller than the proportion of non health-care works who died of the disease.

### Spatial analysis

SaTScan for local cluster detection detects the area of Al Qasim as primary cluster with high rates after adjusting for all explanatory variables (Relative risk(RR) = 1.83, *p* − *value* < 0.0001) and the area of Aseer and Jizan as primary cluster for low rates (RR = 0.093, *p* − *value* < 0.0001) while Al Jawf, Riyadh and Hail were secondary cluster for low rates (RR = 0.51, *p* − *value* < 0.0001). The Wang’s *q*-statistics for global stratified spatial heterogeneity was 0.2285 using the geographical detector method [30, 31]. The spatial stratified heterogeneity analysis indicated no significant stratified spatial heterogeneity of the district MERS incidence (*q* = 0.2285, *p* − *value* = 0.9444).

The estimated posterior odds ratio of mortality from MERS disease and corresponding 95% credibility intervals are shown in Table 2. The results reveal that individuals with comorbidities were twice as likely to have died from MERS-CoV compared with those without comorbidities (OR = 2.071; CI: 1.307, 3.263). Estimates for those individuals that had animal or camel contact, those with secondary contact and results based on gender were not significant. However, individuals who were health-care workers were significantly less likely to have died from the disease compared with non-health workers (OR = 0.372, CI: 0.151, 0.827). Compared with patients who had fatal clinical experience, those with clinical and subclinical experiences were equally less likely to have died from the disease.

**Fig 3:**
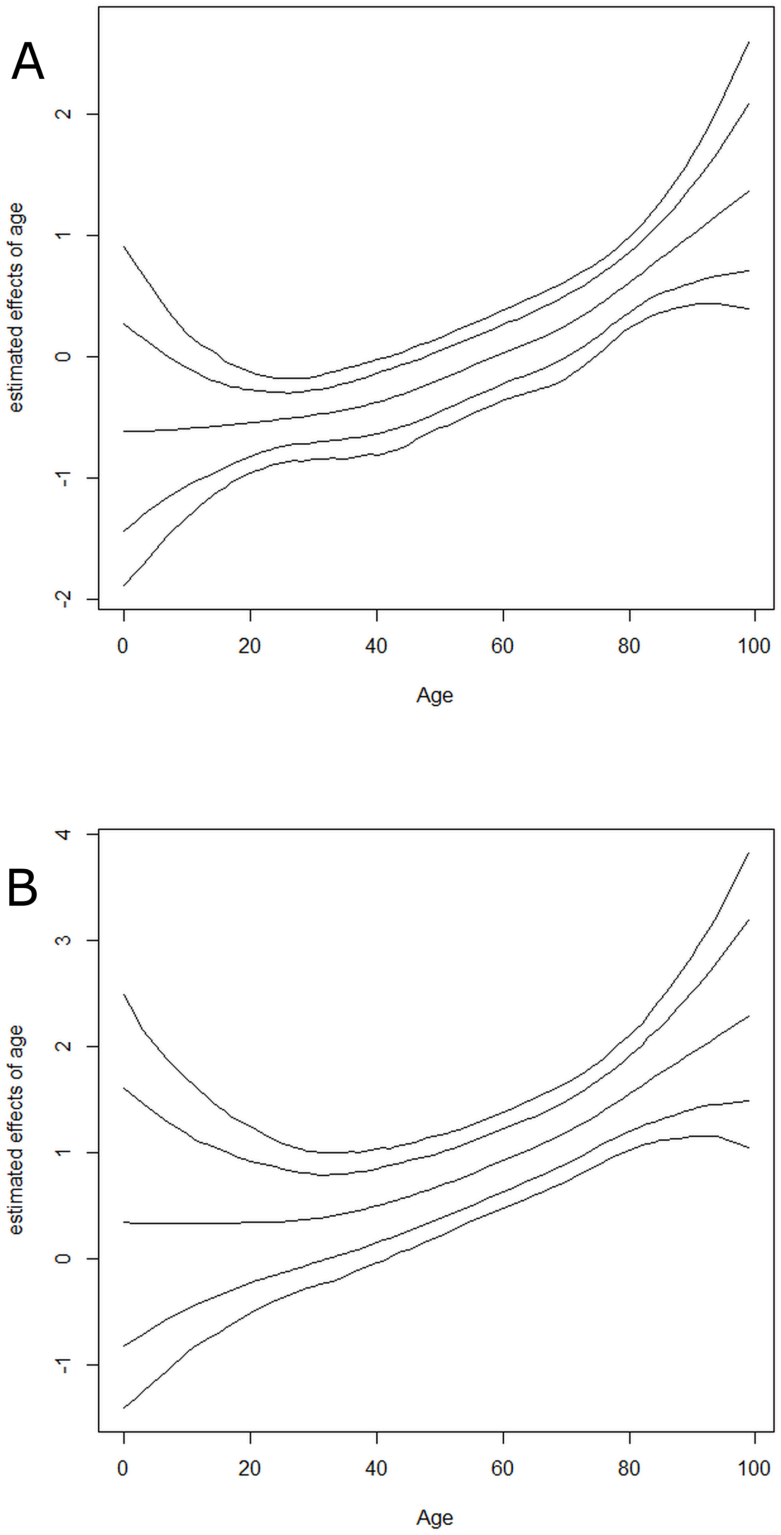
Posterior means of nonlinear effects of age on mortality due to MERS-CoV patients (a) without any underlying comorbidities, (b) with some kind of underlying comorbidities, together with 95% credible interval, adjusted for other covariates.

Fig 3 shows the estimated effects of age (a) and the estimated effects of comorbidity as it varies smoothly over age (interaction between comorbidity and age). Individuals aged 25 years or younger who suffered from MERS-CoV were less likely to have suffered mortality. Nevertheless, the odds of dying from the disease tended to increase as age increased beyond 25 years and was much higher for individuals with any underlying comorbidities.

**Fig 4:**
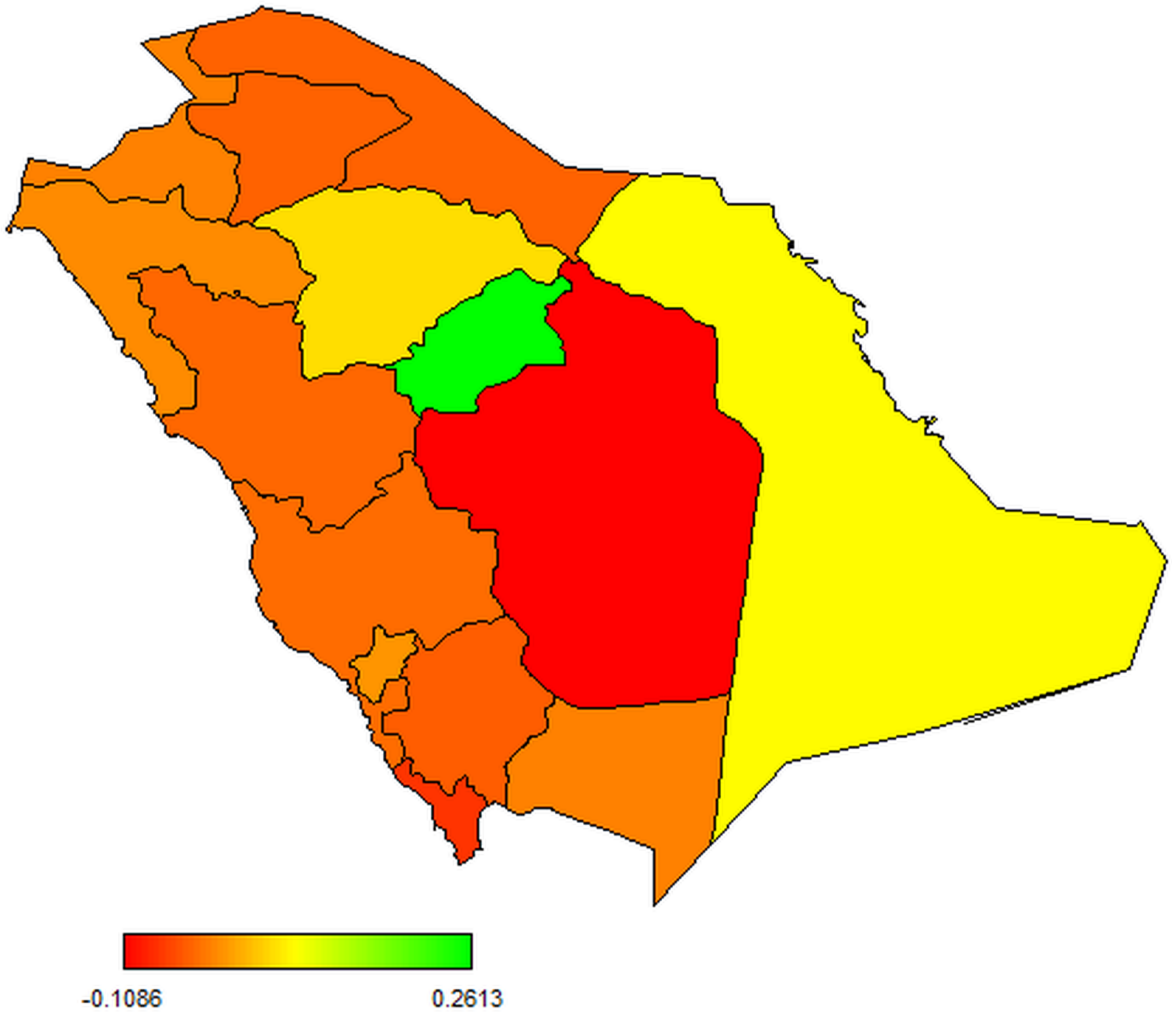
Map showing the posterior means of spatial effects in mortality due to MERS-CoV.

Results of the estimated total spatial variation in mortality due to MERS-CoV are presented in Fig 4. From Fig 4, individuals from provinces with red shading were less likely to have suffered mortality due to MERS-CoV but mortality was higher as the shading moves towards green colour. This implies evidence of significant geographical variation and clustering of mortality from MERS-CoV with lower risk (after adjusting for other variables) occurring in Riyadh, Ar’ar, Al Jawf and Jizan, and higher risk in Al Qasim.

## Discussion

This study that was based on retrospective data of MERS-CoV outbreak in the KSA had 4 main findings. First, patients with comorbidities who were infected with MERS-CoV were as twice likely to die from it than those without comorbidities, after adjusting for confounders. Second, patients with 2 or more comorbidities were more likely to die from MERS-CoV than those with only one comorbidity. Third, health-care workers were 37% less likely to die from the viral infection when compared to non-health care workers. Fourth, our large study sample confirms that individuals under the age of 25, irrespective of comorbidity, and who suffer from MERS-CoV are less likely to die from it, in comparison to the older age groups and that the odds of dying from the disease increased with age.

A number of studies have looked at the epidemiological pattern of the MERS-CoV infection among the community; however, very few have looked at the pattern of deaths among those who are afflicted by the viral disease. The majority of previous studies were limited by the small sample size [12] except two recent ones [14, 24].

Our findings are collectively consistent with the most recent studies on MERS-CoV published by Rivers at al., [24] and Alraddadi et al., [14]. While the work of Rivers and colleagues was practically impeccable, their analysis adopted Poisson regression models using a robust variance estimator without accounting for area-specific geographical effects to capture extra variation in the model. Ignoring spatial pattern in infectious disease may be inadequate to explain the variation in the occurrence of the disease due to space as it has been found that most diseases are location related [41, 42]. Similarly, Alraddadi et al., [14] considered only primary MERS-CoV cases reported in Saudi Arabia during March–November 2014 in their study. They exclude cases with exposure to other cases of MERS-CoV, acute respiratory illness of unknown cause and those exposed to health-care settings within 14 days before illness onset [14].

In our study we adopted the Bayesian spatial modeling to allow for the exact analysis of random effects and coefficient models as well as assess the area-specific spatial effects associated with MERS disease. Assiri’s et al., [12] findings that those with existing health issues are more likely to die from the infection of MERS are also consistent with our findings; nevertheless, the above study only looked at 47 patients with MERS-CoV (28 deaths).

To further strengthen our investigation, we performed a sub-analysis using the “number of comorbidities”, to explore the dose response relationship between comorbidity and mortality from MERS-CoV. The subsequent analysis (univariate) showed that patients who died of MERS-CoV were three times more likely to have one or more comorbidities (OR: 3.4) and almost 5 times more likely to have 2 or more comorbidities (OR: 4.7), compared to patients without any underlying comorbidities. This is a significant finding as it further exposes the negative combined influence of comorbidities on survival, particularly when considering the rise in prevalence of non-communicable disease and the ageing population.

A joint and coordinated worldwide response is unquestionably crucial to tackle new infectious diseases and the threats posed by emerging new strands of viruses that have been able to cause fatal respiratory tract infections over the past decade. These coordinated efforts will optimistically fill major gaps in the understanding of the epidemiology and transmission of the disease. However, these efforts should take place in parallel with the efforts to reduce chronic-non-communicable diseases in our aging population.

The issue of comorbidity is posing further health threats in our time, with chronic and lifestyle diseases on the rise, particularly obesity, diabetes and heart disease. Our research findings and those similar, further warrant the need for effective and successful campaigns to combat chronic illness in the ageing population, not only to reduce mortality and defenselessness against novel and emerging infections, but also to improve the quality of life of these individuals. This is yet another reminder that older and sicker patients are the most vulnerable of all and, thus, require that extra care and watchfulness. Additionally, what makes the situation even more serious is that with today’s unhealthy routine, including occupational and sedentary lifestyle and the abundance of processed and fast foods, more and more people are prone to develop serious comorbidities at a younger age. The Gulf region is indeed not immune to all that as childhood obesity, diabetes and other non-communicable diseases are showing no signs of slowing down. A study from Saudi Arabia showed that more than 50% of Saudi people older than 50 years have diabetes [43]. However, in the studies by Assiri et al., [12] and Mackay et al., [13] the large number of people with MERS-CoV infection and chronic disease might have been due to the hospital outbreak where patients were first admitted. Our results are inline with a recent case-control study where previous medical conditions such as diabetes mellitus, heart disease, and smoking, were each independently associated with MERS-CoV disease [14]. Further case-control studies are needed to define the effect of comorbidities on susceptibility to, and associated mortality from, MERS-CoV infection.

One of the limitations of our study is that it is retrospective rather than prospective. Also, in some cases, infected persons that were admitted for unrelated medical conditions were not considered as having comorbidity and the disease that they were admitted for was not mentioned, although this was not common. There is also the possibility that some patients might have died after discharge; however, this is quite unlikely as patients released from hospital as recovered would have been unlikely to die from the disease without reporting back to the hospital and medical team when health deteriorated. Lastly, because in some cases patients history and contacts with animals or camel cannot be verified, there is a possibility of patients giving false or inaccurate information.

## Conclusion

This study has revealed that individuals with comorbidity, non-health-care workers, patients with no clinical fatal experience, and patients without any clinical and subclinical experiences significantly increased the odds of death from MERS-CoV in the Arabian Peninsula. It is therefore imperative for public health practitioners, policy makers and program managers to principally target these individuals when they are formulating and implementing strategies to minimize deaths from this syndrome. More work should be done to treat and prevent multiple comorbidities, particularly within the aging population, in order to lessen the risk of death when the individual is hit by a new and emerging disease.
